# Hydrochemical Analysis and Fuzzy Logic Method for Evaluation of Groundwater Quality in the North Chengdu Plain, China

**DOI:** 10.3390/ijerph16030302

**Published:** 2019-01-23

**Authors:** Adam Khalifa Mohamed, Dan Liu, Kai Song, Mohamed A. A. Mohamed, Elsiddig Aldaw, Basheer A. Elubid

**Affiliations:** 1 Faculty of Geoscience and Environmental Engineering, Southwest Jiaotong University, Chengdu 611756, China; adamkh124@yahoo.com (A.K.M.); songkailw@163.com (K.S.); siddigeldaw114@yahoo.com (E.A.); elubaid@yahoo.com (B.A.E.); 2 Faculty of Water and Environmental Engineering, Sudan University of Science and Technology, Khartoum 11111, Sudan; insiab2@yahoo.com

**Keywords:** groundwater quality, hydrochemical facies, WHO standard, fuzzy logic, North Chengdu plain

## Abstract

Groundwater is a major water resource in the North Chengdu Plain, China. The research objective is to determine the quality and suitability of groundwater for drinking purposes within the vicinity of a shallow, unconsolidated aquifer of Quaternary age. In this study, a detailed investigation was conducted to define the hydrochemical characteristics that control the quality of groundwater, based on traditional methods. Considering the uncertainties linked with water resources and the environmental complications, the fuzzy logic method was used in the determination of groundwater quality for more precise findings that support decision-making. To achieve such an objective, sixteen water quality guidelines were used to determine groundwater quality status in six selected wells. The results showed that the groundwater is neutral, very hard, and fresh in nature. Dominating cations and anions are in the order of Ca${}^{2+}$ > Na${}^{+}$ > Mg${}^{2+}$ > K${}^{+}$ and HCO${}_{3}^{-}$ > SO${}_{4}^{2-}$ > Cl${}^{-}$. The Piper trilinear diagram demonstrates that the hydrochemical facies of groundwater are mostly of Ca-HCO${}_{3}$ type. Statistical analysis denotes a positive correlation between most of the chemical parameters. The study took the results of the fuzzy logic evaluation method into consideration, to classify the samples into five groups according to the Chinese groundwater quality standard (GB/T 14848-93) for their suitability for domestic use. The results demonstrated that the quality of the groundwater samples is within grade II and III, and is suitable for drinking purposes. The comprehensive evaluation of groundwater quality is critical to aid sensitive policy decisions, and the proposed approach can guarantee reliable findings to that effect. The results of this study would also be helpful to future researches related to groundwater quality assessment.

## 1. Introduction

One of the fundamental sources of water for drinking, irrigation, and industrial use in many countries is groundwater [1,2]. Recently, environmental protection and water resource quality management have become paramount issues in designing policies for public health [3,4]. The contamination of groundwater is a major problem, which has posed serious threats to human health and environmental quality worldwide [5]. The issue of groundwater contamination is caused by various human activities such as agricultural, industrial, and others, leading to infiltration of nitrates, organic matter, and pesticides, among others, deep into the aquifer [6]. The chemical property of natural groundwater is generally perfect, however, environmental factors associated with the increase in concentrations of some components can cause complications when it comes to using the water [7,8]. With accelerated development, coupled with the indisciplined approach in the execution of some development projects, groundwater is impacted continuously by agriculture, mining, industry, and other anthropogenic activities [9,10]. Waste products are discharged into the river systems resulting in the pollution of both surface and groundwater [6,11]. However, these systems often do not have leakage prevention, resulting in direct or indirect infiltration of wastewater into local aquifers and subsequent contamination of the groundwater resources [5].

Once groundwater is polluted, its quality cannot be reinstated by halting the contaminants from their sources. Therefore, it is necessary to regularly observe the standard of groundwater and formulate ways to protect it [7] as the quality of water is a critical factor for human health. This is the case of shallow aquifer in the quaternary unconsolidated sedimentary basin, which is considered to be the largest source of drinking water in the North Chengdu Plain, China. This situation has challenged the managers of water resources, and has focused their attention on the evaluation of the contamination of groundwater in the study area based on more robust assessment methods. The appraisal of groundwater quality is often founded on hydrochemical analysis, which provides much information about the water samples [12]. Also, hydrochemical analysis is very useful for analyzing a lot of actual problems, such as study of mixed waters from different sources, groundwater quality condition, the effect of various structures on water quality, and inspection of the origins of salinity [13].

The traditional techniques, such as descriptive statistics, stiff pattern diagrams, semi-logarithmic graphs, Piper trilinear diagrams, and others, are widely used for analyzing the water samples [14,15,16,17,18]. These techniques focus on the survey of natural concentrations of ions and metals in groundwater. They also focus on the assessment of their suitability for different purposes by comparing the measured quality parameters with predefined standards parameters [19]. Among these methods, descriptive statistics, Piper trilinear diagrams, and Person’s correlation have been used in this work. These approaches are useful in defining the hydrochemical characteristic of groundwater. Also, they are deemed appropriate methods to avoid misinterpretation of environmental monitoring data [20,21]. Therefore, we apply these techniques to provide descriptive statistics for preliminary information about the water quality. This information constitutes a necessary stage in the complete evaluation of the water quality and serves as important tool for classification and interpretation of the monitoring data [22]. By descriptive statistics, the groundwater suitability for domestic purposes was assessed by comparative analysis of the water quality parameters against the benchmark parameters in the WHO (2011) and Chinese national (GB 5749-2006) standards. The Piper trilinear diagram has been employed for geochemical evaluation of the groundwater samples, to determine the hydrochemical facies of groundwater [12]. Pearson’s correlation coefficient was used to reveal the connection between physiochemical parameters at each site.

Several methods, such as gray correlation analysis, neural network model, set-pair analysis, entropy method, water pollution index, statistical analysis, and others have been used to assess groundwater quality [5]. The analysis approaches were chosen to suit the specific study objectives [23]. However, most of the methods mentioned above are deterministic, and the linear relationship between the water quality assessment variables may affect the results [24]. Also, many of these methods employed for the groundwater quality assessment cannot directly reflect pollution characteristics. As a result, some inaccuracies and ambiguities during decision making, concerning the boundary values are experienced [25]. In the same vein, the deterministic methods are less viable and are challenging to popularize in the regional groundwater pollution evaluation. This is due to complex and unpredictable environmental problems [24]. The fuzzy logic method is an artificial intelligence approach, which provides a useful technique to deal with the imprecision in classification based on the water quality standards [26]. Globally, the fuzzy logic method has become widely employed in groundwater studies [27]. It has a large range of classification capabilities, which can be applied to identify and overcome the uncertainties regarding the class boundary for groundwater quality grading. Additionally, the fuzzy logic method has been recommended in the literature of water quality assessment to deal with the boundary uncertainty problems and to control the effect of monitoring errors on evaluation results [26,27,28,29].

In order to attain reliable results for concise decision-making regarding the uncertainty associated with the complex environmental risks, artificial intelligence approaches, such as the fuzzy logic method, together with in-depth statistical analysis, has been adopted in the current study to assess the quality of groundwater in the research background. Specifically, we apply fuzzy logic to evaluate/categorize groundwater from six selected wells in the North Chengdu Plain, based on the Chinese national standard. The combination of fuzzy and the traditional methods has the potential to mitigate the shortfalls associated with the traditional methods for concise water quality decision-making. Traditional techniques are inadequate to classify or measure environmental effects of an inherent nature or even offer ways to handle the lost data. Fuzzy logic is capable of combining these different techniques, because it is highly efficient and reliable universally [25]. Furthermore, the basic information about groundwater standards in the study area, which could be of assistance in understanding the hydrochemical properties of the groundwater quality in the study, is ascertained by the traditional approach.

## 2. Materials and Methods

### 2.1. Study Area Description

**Figure 1:**
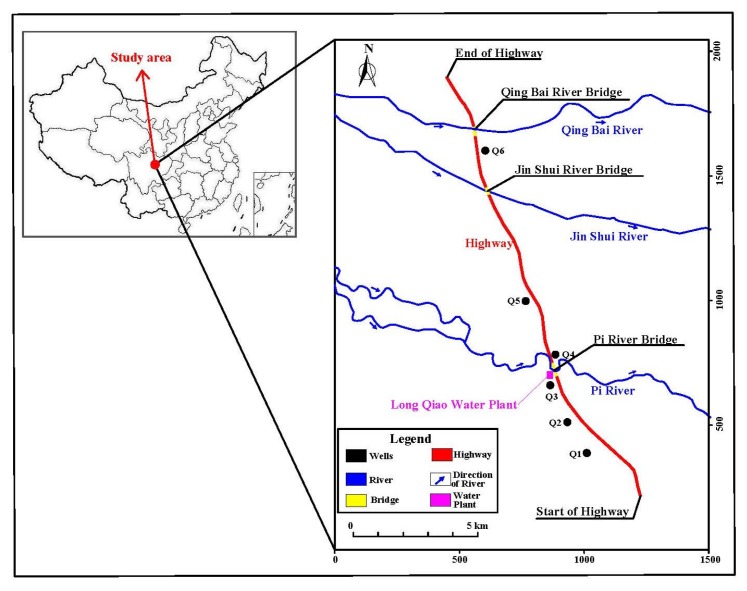
Study area site and groundwater samples in the North Chengdu Plain.

The study region is situated in the North Chengdu Plain of Sichuan Province, China. This area comprises many villages with a population of more than 140,000. It is bounded between longitudes $103^{\circ }54^{\prime }02^{\prime \prime }$ to $104^{\circ }16^{\prime }54^{\prime \prime }$ and $30^{\circ }40^{\prime }40^{\prime \prime }$ to $30^{\circ }57^{\prime }58^{\prime \prime }$. The region is approximately 10 km away from Chengdu City (Figure 1), and it is has a total area of 18.077 km${}^{2}$. The groundwater from the Quaternary unconsolidated sediments is the main source of water for domestic and other purposes in the study area. This basin gives the highest amount of water for the residents. The domestic sewage water, industrial waste water, and the use of chemical fertilizers for agriculture are considered as the main sources of groundwater pollution in this region. All these sources are discharged into the nearby rivers through the sewer systems. We believe that most sources of groundwater contamination are infiltrated into the underlying aquifer, due to persistent rains and the shallowness of the aquifer. Therefore, under such conditions, the quality of groundwater would be particularly important, and it is necessary to assess its quality in the study area due to the linkage between water quality and peoples’ health. The climate in this region is sub-humid monsoon, which comprises four seasons. The monthly average of cold and hot temperature is 4.6 ${}^{\circ }$C and 24.4 ${}^{\circ }$C respectively with annual mean value of 10.4 ${}^{\circ }$C. Rainfall is the main recharge source in this area, with an annual average of about 1134.8 mm.

The study region is in the hinterland of Chengdu Plain; the plain is based on the syncline which goes from northeast to southwest and is formed by the Minjiang River, the Tuojiang River, and their tributaries, as well as their alluvial fan. The quaternary loose in the ground of the plain, whose top is covered with silt and clay sediments, has a large thickness. The ground in the area is flat, and its altitude is between 450 to 750 m. The overall terrain is tilting from northwest to southeast with the average gradient of 3 to 10%. The relative height difference in the area is always less than 20 m. The soil is mostly brown silty clay, and the geological structure of plain consists of hard Cambria pegmatite fundus with miscellaneous fill of low depth about 5 to 7 m, and a sand-cobble layer of average depth about 7 to 10 m. Since the looseness is of different fill, sand, and cobble, there are dense small gaps within the structure, thus enabling the absorption of travelling seismic waves [30]. The main type of aquifer in this area is unconsolidated Quaternary sediments, with groundwater table depth varying between 1.5 and 6 m. The general groundwater flow direction in the aquifer is from northwest to southeast.

### 2.2. Sampling Procedures and Analysis

In this work, groundwater samples were monitored from six wells chosen from the North Chengdu Plain. The wells are: Shi Qiao village well (Q1), Chang He village well (Q2), LongQiao water work original water well (Q3), Qing Qiao village well (Q4), Hua Hong village well (Q5), and Qingbai village well (Q6). Figure 1 represents an applied methodology for sampling. It is worth mentioning that a comprehensive field survey was conducted in the study area, and it was observed that the locations of the wells are exposed to the risk of groundwater pollution. This situation is the basis for choosing these sites for the research, due to their relative significance in the study area as a source of drinking water. The samples collected were analyzed for sixteen different physicochemical water quality parameters (which include pH, EC, TH, TDS, Na${}^{+}$, K${}^{+}$, Ca${}^{2+}$, Mg${}^{2+}$, HCO${}_{3}^{-}$, Cl${}^{-}$, SO${}_{4}^{2-}$, NO${}_{2}^{-}$, NO${}_{3}^{-}$, NH${}_{4}^{+}$, Mn, and Fe), in the laboratory of Southwest Jiaotong University during January to December 2017. It should be noted that, for each well, four samples were taken in January, April, August, and December respectively; there was no apparent change in the analysis results of these samples and therefore it was taken as the average in this study. After 10 minutes of pumping, these samples were collected and stored in polyethylene bottles, that had been washed two or three times with the groundwater, to be analyzed. All the bottles were accurately labeled and numbered before being transported and kept in a refrigerator at a temperature below 4 ${}^{\circ }$C until analyzed. The physiochemical parameters, such as pH, electrical conductivity (EC), and total dissolved solids (TDS), were taken in-situ because they may change through transportation. The groundwater samples have been analyzed for the technical parameters, which are considered to be an indicator of groundwater contamination by domestic and industrial wastewater discharges. The technical parameters comprise hydrogen ion concentration (pH), electrical conductivity (EC), total hardness (TH), and total dissolved solids (TDS). Adding to them, important cations such as sodium (Na${}^{+}$), potassium (K${}^{+}$), calcium (Ca${}^{2+}$), and magnesium (Mg${}^{2+}$), as well as anions such as bicarbonates (HCO${}_{3}^{-}$), chlorides (Cl${}^{-}$), sulfates (SO${}_{4}^{2-}$), secondary ions such as ammonia (NH${}_{4}^{+}$), nitrite (NO${}_{2}^{-}$), nitrate (NO${}_{3}^{-}$), and heavy metals (Mn and Fe). The samples were analyzed in the laboratory using standard analytical methods, as per BIS and the American Public Health Association guidelines [31]. Table 1 shows the different analysis parameters of the sampled water with their applicable methods.

### 2.3. Evaluating Groundwater Quality by the Fuzzy Logic Method

**Figure 2:**
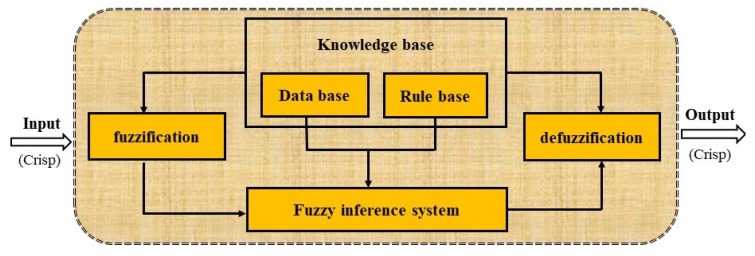
Schematic of the methodology for a groundwater sustainability index.

Undertaking a risk assessment of groundwater contamination is often challenging because it involves interpreting many groundwater quality parameters. Fuzzy logic, proposed by Zadeh in 1965 as a new way to represent vagueness in everyday life [25], can simplify this risk assessment process because it takes fuzzy mathematics as the foundation to transform the uncertain border factors into certain ones [32]. It has been applied throughout the world in decision-making and assessment processes in an inaccurate environment, and the result is more actual and satisfactory [26]. According to the literature [29,33,34], the basic idea in water quality assessment by fuzzy logic evaluation method consists of four operations: First is the fuzzification process, in which crisp values of input and output variables are converted into fuzzy values to compare measured concentration of evaluation factors with the standard value to calculate the subordination degree and establish the fuzzy relation matrix. In the second knowledge base operation, the database and rule base define the weight of each factor and establishes a weight coefficient matrix. The core section of a fuzzy logic system, also known as the decision-making unit, performs the inference operations on the rules in the third phase of the operation. Finally, defuzzification is performed as a composite operator, which operates the fuzzy relation matrix together with the weight coefficient matrix and obtains the fuzzy comprehensive evaluation matrix to a quality of water in all categories, which is then transformed into a crisp number. Following the principle of maximum membership degree, the grade corresponding to the maximum value of the comprehensive degree of membership is the grade to which the water quality belongs to. Figure 2 shows the schematic diagram of the overall process of construction and implementation of the fuzzy logic system. Details of the steps of fuzzy logic evaluation are described as follows.

#### 2.3.1. Establish the Evaluation Factors and Determine the Standard Value

The principal procedure of fuzzy logic evaluation method includes establishing the evaluation factors U = $\left(u_{1},u_{2},u_{3},\ldots ,u_{n}\right)$. The elements $u_{i}\left(i=1,2,\ldots ,n\right)$ represent the measured values of pollutants affecting the groundwater’s quality. The quality evaluation grades of water $V=(v_{1},v_{2},\ldots ,v_{m})$ are represented by $v_{i}\left(i=1,2,\ldots ,m\right)$, and represent the evaluation standard sets corresponding to u${}_{i}$. The Chinese national standards of groundwater quality (GB/T 14848-93) has been used in this paper as a standard of evaluation. The standard mentioned above is issued from the Ministry of Geology and Mineral Resources of the Peoples’ Republic of China. The quality of groundwater is classified into five categories by the standard (GB/T 14848-93). These five categories are grade I, grade II, grade III, grade IV, and grade V, established in accordance with the quality of groundwater in China land and the requirements for human health under the constraint of protecting the water. The contamination of groundwater includes five different levels according to the standard (GB/T 14848-93). These five levels are: Grade I–excellent, grade II–good, grade III–moderate, grade IV–poor, and grade V–very poor [35], as displayed in Table 2.

#### 2.3.2. Membership Degree and Construction of Fuzzy Matrix R

The membership function characterizes the fuzzy set entirely, and this can be expressed in various forms, such as trapezoidal, triangular, Gaussian, pseudo-exponential, sigmoidal, and so on [36]. The membership function links each point in a fuzzy set to a membership grade between 0 and 1 [37]. The fuzzy set describes the relationship between a membership function (μ) and an uncertain quantity (*x*). The value of the membership qualifications increases when the membership’s value increases. For instance, the membership is subordinated completely when its value equals 1. In contrast, the membership is considered not subordinated entirely when its value equals 0. The fuzzy set’s membership degree is determined over the *X* domain, taking the following form:

(1) $$\mu_{A}:X\to \left[0,1\right].$$

The fuzzy set *A* is a subset of the universe of discourse *X*, which is denoted by the following nomenclature:

(2) $$\mu_{A}\left(x\right)=\left\{\left(\mu_{A}\left(x\right)\right),x\in X,\mu_{A}\left(x\right)\in \left[0,1\right]\right\},$$

where *x* belongs to *X* and is an element of fuzzy set *A*, and the value of $\mu_{A}\left(x\right)$ shows the membership grade of *x* in fuzzy set *A*.

In this paper, we have employed the trapezoidal function of the membership in order to normalize the inputs of the crisp. According to [34], the trapezoidal membership function is suitable due to its simple structure and high computational efficiency. The trapezoidal membership function can be represented mathematically for groundwater quality parameters, taking into account grade I, grade II, grade III, grade IV, and grade V classifications, in the following equation [38].

(3) $$\mu_{A}\left(x\right)=\left\{\begin{array}{cc} 0, & x<\alpha \\ \frac{x-\alpha }{\beta -\alpha }, & \alpha \leq x\leq \beta \\ 1, & \beta \leq x\leq \gamma \\ \frac{\delta -x}{\delta -\gamma }, & \gamma \leq x\leq \delta \\ 0, & x>\delta . \end{array}\right.$$

For a trapezoidal membership function, $\mu_{A}\left(x\right)$ represents the membership value, δ is the maximum value, α is the minimum value, and β and δ are the two values which represent the interval of the most likely value.

The fuzzy evaluation matrix *R* is the comprehensive survey to the index of safety evaluation of groundwater, and it reflects the membership of the *j*-th water quality category corresponding to the *i*-th water quality index, as represented in Equation (4).

(4) $$R=r_{ij}=\left[\begin{array}{cccc} r_{11} & r_{12} & \cdots & r_{1m}\\ r_{21} & r_{22} & \cdots & r_{2m}\\ \vdots & \vdots & \vdots & \vdots \\ r_{ni} & r_{n2} & \cdots & r_{nm}, \end{array}\right]$$

where $r_{nm}$ stands for the membership degree of *n* (evaluation factor) to *m* (water category, 1≤m≤5).

#### 2.3.3. Weights Coefficient Matrix

The factor weights are essential in the mathematical model of fuzzy logic. The weighting coefficient of pollution factor is a measure of assessing various contaminant factors effect on the groundwater quality. The weight calculation is achieved by weighting the degree to which the single factor exceeds the standard grades. The weight ($W_{i}$) of the groundwater quality factor is calculated from the following equation [39]:

(5) $$W_{i}=\frac{C_{i}}{S_{i}},$$

where $W_{i}$ is the weight of the evaluation factor *i*, $C_{i}$ is the actual concentration of the evaluation factor *i*, and $S_{i}$ is the concentration limits of relevant factors of different categories $\left(S_{i}=\left(S_{1}+S_{2}+S_{3}+S_{4}+S_{5}\right)/5\right)$. The normalized weight of each factor is calculated from the following equation.

(6) $$a_{i}=\frac{C_{i}/S_{i}}{\sum_{i=1}^{n}\frac{C_{i}}{S_{i}}}=\frac{W_{i}}{\sum_{i=1}^{n}W_{i}}\;\;\;\;\;\;\;\left(1\leq i\leq n\right),$$

where $a_{i}$ is the normalization of the evaluated factor *i* and $\sum_{i=1}^{n}W_{i}$ is the summation of the weight in all groundwater quality parameters. According to Equation (6), the single factor weight set can be obtained as $A=\{a_{1},a_{2},\ldots ,a_{i}\}$.

#### 2.3.4. Fuzzy Comprehensive Evaluation Matrix

The groundwater quality assessment by fuzzy logic membership is based on the matrix *B*, which is obtained by matrix multiplication of the weight coefficient matrix *A* and fuzzy relation matrix *R* of the grades I, II, III, IV, and V separately [40]. The equation for the evaluation matrix is given as follows:

(7) $$B=A\times R=\left\{a_{1},a_{2},\ldots ,a_{i}\right\}\times \left[\begin{array}{cccc} r_{11} & r_{12} & \ldots & r_{1m}\\ r_{21} & r_{22} & \ldots & r_{2m}\\ \vdots & \vdots & \vdots & \vdots \\ r_{ni} & r_{n2} & \ldots & r_{nm} \end{array}\right]=\left\{b_{1},b_{2},\ldots ,b_{m}\right\}.$$

The fuzzy *B* is the matrix of the membership of each groundwater quality class. The groundwater sample is classified in the class with maximizing membership.

## 3. Results and Discussion

### 3.1. Hydrochemical Characteristics of Groundwater

#### 3.1.1. Descriptive statistics method

The different physicochemical parameters of groundwater samples were analyzed, and the statistical parameters such as minimum, maximum, mean, and standard deviation concentration of physicochemical parameters are shown in Table 3. In this study, the drinking water standards of the WHO (2011) and Chinese national (GB 5749-2006) standards form the basis for the evaluation of groundwater quality for drinking purposes.

**Figure 3:**
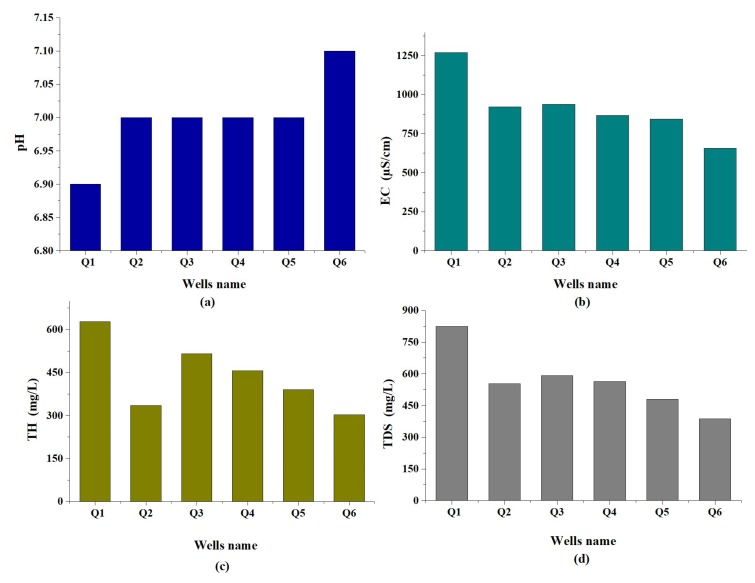
Spatial variation in (**a**) pH, (**b**) EC, (**c**) TH and (**d**) TDS of groundwater sample in the study area.

The concentration of all physicochemical parameters of groundwater in the study area are discussed below:

**pH**: The pH of water provides biotic information in numerous types of geochemical equilibria or solubility calculations, and indicates the strength of the water to react with the acidic or alkaline material present in the water [41]. If the value of pH ranges from 7 to 14, the sample considered is alkaline, it is acidic if the pH varies from 0 to 7, and it is neutral when the pH equals 7. According to the WHO (2011) standard guidelines and GB 5749-2006, the pH values for drinking water ranges between 6.5 to 8.5 [42,43]. The water sample pH values varied from 6.9 to 7.1, with 7 as the mean value. This clearly indicates that the groundwater in the area of study is neutral in nature for most of the groundwater samples, as shown in Figure 3a. Therefore, the pH is within the permissible limit of WHO 2011 and the Chinese national standard (GB 5749-2006) for drinking water.

**Electrical conductivity (EC)**: The ability of electrical current in the water is demonstrated in the electrical conductivity as an outstanding quality of groundwater parameters. This parameter is a function of the existence of ions and has a direct relationship with the total dissolved solids (TDS) [44]. The values of the electrical conductivity were within the interval 655.93–1269.08 μS/cm, while the mean value was 915.75 (Figure 3b). We have noted that the entire groundwater samples were found to be above the permissible limit of WHO 2011 (500 μS/cm). The higher value in electrical conductivity was mainly attributed to mineral dissolution and anthropogenic influence (as well as agricultural activities), causing an increase in the concentration of ions [45]. The electrical conductivity is categorized as type I if the enrichment of salts is lower than 1500 μS/cm. The EC is Type II when the enrichment of salts varies from 1500 to 3000 μS/cm, and it is considered type III when the salt enrichment is higher than 3000 μS/cm. According to this category, all the groundwater samples derived from the study area were of type I (low enrichment of salts).

**Total hardness (TH)**: It was found that the total hardness (TH) is an important parameter to evaluate water quality if it is suitable to be used in domestic, agricultural, or industrial purposes. The hardness values ranged from 302.80 to 628.10 mg/L, with an average of 437.92 mg/L (Figure 3c). Based on the hardness [46], groundwater exceeding the limit of 300 mg/L is considered to be very hard, 150–300 mg/L to be hard, 75–150 mg/L to be moderately hard, and <75 mg/L to be soft. According to the above classification, the total groundwater samples fell into ‘very hard’ category. Therefore, the present study reveals that all samples exceeded the permissible limit of (WHO 2011). According to the Chinese national standard (GB 5749-2006), 50% of the samples had TH value which exceeded the permissible limit of 450 mg/L, as represented in the sampling sites Q1, Q3, and Q4, with the others remaining within the permissible limit. The water hardness was caused by the presence of alkaline piles of the earth, such as calcium and magnesium. The higher value of TH was mainly attributed to leaching of Ca${}^{2+}$ and Mg${}^{2+}$ ions into groundwater, and anthropogenic activities. High TH for the long-term consumption of extremely hard water might lead to increased occurrence of urolithiasis, anencephaly, prenatal mortality, and some varieties of cancer-related cardiovascular disorders [47].

**Total dissolved solids (TDS)**: TDS illustrate the groundwater salinity behavior, and ranged between 387 to 824.90 mg/L, with a mean value of 566.63 mg/L (Figure 3d). The higher permissible limit for TDS values for drinking water is 500 and 1000 mg/L, according to WHO (2011) and the Chinese national standard (GB 5749-2006) respectively.

In the present study, two sampling sites, Q5 and Q6, of the total number of samples are within the permissible limit, recommended by the WHO (2011) of 500 mg/L, and the others exceeded the permissible limit. This observation may be attributed to agricultural practices, leaching of salts from the soil, and anthropogenic activities. With the Chinese national standard (GB 5749-2006), the values of TDS obtained for almost all the groundwater samples collected in this study are within the permissible boundary. By using TDS, the groundwater quality degree is categorized into: Fresh, when the TDS is less than 1000 mg/L; brackish, when TDS is in the range 1000–10,000 mg/L; saline, if within the range 10,000 to 1,000,000 mg/L; and brine, if the TDS is more than 1,000,000 mg/L [48]. According to this classification, the quality of all of the groundwater in the study area was classified as freshwater type.

**Figure 4:**
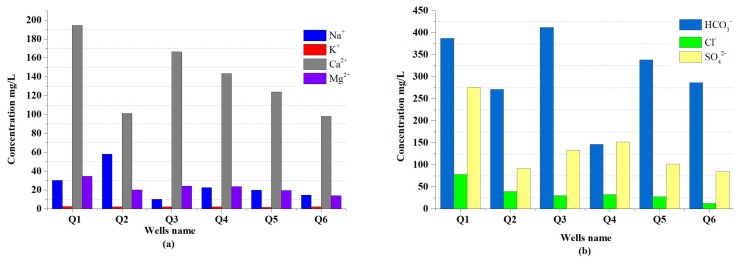
The spatial allocation of (**a**) cations (Na${}^{+}$, K${}^{+}$, Ca${}^{2+}$, and Mg ${}^{2+}$) and (**b**) anions (HCO${}_{3}^{-}$, Cl${}^{-}$, and SO${}_{4}^{2-}$) in the study area.

**Sodium and Potassium (Na${}^{+}$, K${}^{+}$)**: The values of cation concentrations Na${}^{+}$ and K${}^{+}$, at each sampling well, are shown in Figure 4a. Sodium is the major dominant ion in the cation chemistry. The concentration of sodium ions Na${}^{+}$ in the groundwater samples collected varied from 10.20 to 58.0 mg/L, with an average value of 25.95 mg/L. The concentration of K${}^{+}$ ranged between 1.80 to 2.50 mg/L, with a mean value of 2.25 mg/L in the groundwater of the study area. As per WHO (2011) standard guidelines and Chinese national standard (GB 5749-2006), the permissible limit for drinking water is 200 and 12 mg/L of sodium and potassium, respectively. In this study, the concentrations of Na${}^{+}$ and K${}^{+}$ were found to be below the permissible limit of both standards.

**Calcium and Magnesium (Ca${}^{2+}$, Mg${}^{2+}$)**: Calcium and magnesium are often present in significant concentrations of natural water and are directly related to hardness. The principal sources for the elements, in most of the samples of groundwater, include detrital minerals such as plagioclase feldspar, pyroxene, amphibole, and garnet [49]. According to [50], calcium is an essential nutrient for living things. It’s deficiency lead to rickets. In our study, the concentration of calcium ions Ca${}^{2+}$ took values between 98.20 to 194.40 mg/L, with an average value of 137.90 mg/L, as shown in Figure 4a. In all samples of groundwater obtained, the concentration of calcium ion was above the permissible limit of WHO (2011) (75 mg/L). High calcium ion concentrations may provoke abdominal diseases, and it is not recommended to be used for domestic purposes, as it causes encrustation and scaling. On the other hand, the concentration of magnesium ions Mg${}^{2+}$ ranged from 13.98 to 34.66 mg/L, with mean value of 22.72 mg/L, as illustrated in Figure 4a. The magnesium concentration in all the groundwater samples was found within the WHO (2011) permissible limit of 50 mg/L.

**Bicarbonate (HCO${}_{3}^{-}$)**: The spatial distribution of anion bicarbonate in the study area is given in Figure 4b. The primary source of the groundwater’s HCO${}_{3}^{-}$ anion comes from mineral dissolution—for instance, dolomite and calcite—and it is responsible for the alkalinity of groundwater [51,52]. The value of HCO${}_{3}^{-}$ was observed from 146.40 to 411.90 mg/L, with an average value of 307.12 mg/L. The total number of samples are within the permissible limit of 500 mg/L, as recommended by the WHO (2011).

**Chloride (Cl${}^{-}$)**: In the groundwater, chloride ion concentration emerges typically from sources such as solubility of chloride-bearing evaporation deposits and anthropogenic sources. High concentrations of Cl${}^{-}$ gives a salty taste to water and may result in hypertension, osteoporosis, renal stones, and asthma [53]. The chloride ion (Cl${}^{-}$) concentration in the collected groundwater samples varied from 12.79 to 78.15 mg/L, with a mean value of 36.86 mg/L (Figure 4b). Chloride content was found lower than the accepted limit of 250 mg/L at all the sampling sites, according to WHO (2011) and Chinese national standard (GB 5749-2006).

**Sulphate (SO${}_{4}^{2-}$)**: Sulphates are originated from natural sources, as well as industrial effluents. The sulphate concentration in the study area was observed to be from 85.03 to 276 mg/L, with an average of 140.04 mg/L, as shown in Figure 4b. The higher limit of sulphate concentration for drinking water is 250 mg/L, from the WHO (2011) and Chinese national standard (GB 5749-2006). The present study reveals that one sampling site (Q1) of the total number of samples exceeded the permissible limit of both standards. The high concentration of sulphate in groundwater resulted due to contamination of untreated industrial and domestic waste and their effluents, as well as precipitation, solution, and concentration of the traverse via the sedimentary rock, such as anhydrite and gypsum [54].

**Figure 5:**
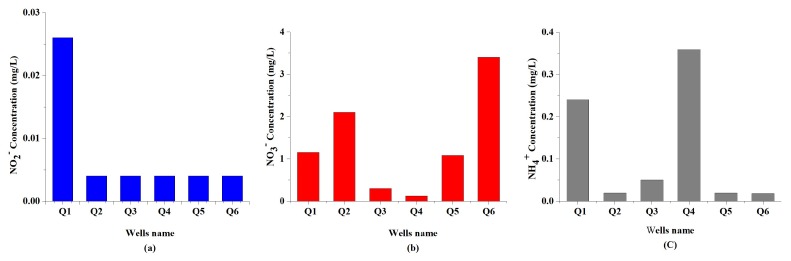
The values of concentrations (**a**) NO${}_{2}^{-}$, (**b**) NO${}_{3}^{-}$ and (**c**) NH${}_{4}^{+}$ of groundwater samples in the study area.

**Nitrite and Nitrate (NO${}_{2}^{-}$, NO${}_{3}^{-}$)**: The most widespread contaminants are nitrogen compounds in sub-surface areas, mainly originated from decaying organic matter, leakage of septic tanks, sewage wastes, and fertilizers, as well as the infiltration of nitrate with the leaching water [55]. Nitrite happens as an intermediary product of the transfer of ammonium ion to the nitrate, likewise due to the process of nitrification of ammonia [56]. The concentration of nitrite, as shown in Figure 5a, varied from 0.004 to 0.03 mg/L, with an average of 0.01 mg/L. These values are within the WHO (2011) prescribed limit and Chinese national standard of 3.0 and 0.02 mg/L, respectively, excluding the value of the sample Q1 which is higher than the permissible limit of Chinese national standard. Nitrite can oxidize hemoglobin to methemoglobin and hinder the transportation of oxygen around the body. Therefore, nitrite is more harmful than nitrates in the drinking water supply [57].

Nitrate content in the study area was in the range of 0.12 to 3.4 mg/L, with an average value of 1.36 mg/L (see Figure 5b). It was found to be within the prescribed limit of all groundwater samples. The presence of high NO${}_{3}^{-}$ in the drinking water might lead to methemoglobinemia in infants, goiter, birth malformations, hypertension, and some types of gastric cancer disorders [7].

**Ammonia (NH${}_{4}^{+}$)**: Ammonia is considered one of the rare minerals in natural water, but its existence is quite general as a result of natural decomposition of organic matter. Sewage water has significant quantities of nitrogenous matter. Hence, its disposal tends to increase the ammonia content of water [58]. The ammonia in groundwater samples varied from 0.02 to 0.36 mg/L, with an average of 0.12 mg/L, as shown in Figure 5c. The values are much less than the permissible limits as described by WHO (2011) guideline value for total ammonia in drinking water. The Chinese national standard described the samples of wells Q1 and Q4 out of range of the permissible limit, whereas the remaining wells are within the limit.

**Figure 6:**
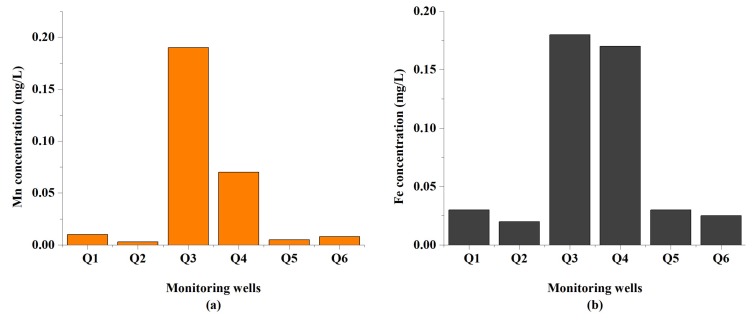
Spatial distribution of concentrations (**a**) Mn and (**b**) Fe in the study area.

**Iron and Manganese (Fe, Mn)**: Iron is one of the elements available in the rocks and soil, which is found in considerable quantities in all kinds of waters. Even though the metal has received little attention as a health hazard, it is still considered a nuisance when it exceeds quantities of both domestic and industrial uses [50]. In the study area, Fe was obtained in the range from 0.02 to 0.18 mg/L, with an average of 0.08 mg/L, as shown in Figure 6a.

The manganese concentration (Mn) was observed from 0.003 to 0.19 mg/L, with a mean value of 0.05 mg/L. From the samples analyzed, all samples of groundwater of Fe and Mn are found to be less than the permissible limit set by WHO (2011), and Chinese national standard (GB 5749-2006), except sample Q3, which exceeds both standards for Mn. In the basin Quaternary unconsolidated sedimentary of the North Chengdu Plain, an amount of Mn is detected in the sediment by considering the conditions of a natural environment [5]. Therefore, the increase of Mn is considered natural. The spatial distribution of the manganese ion in the study area is shown in Figure 6b.

#### 3.1.2. Hydrochemical Facies of Samples

**Figure 7:**
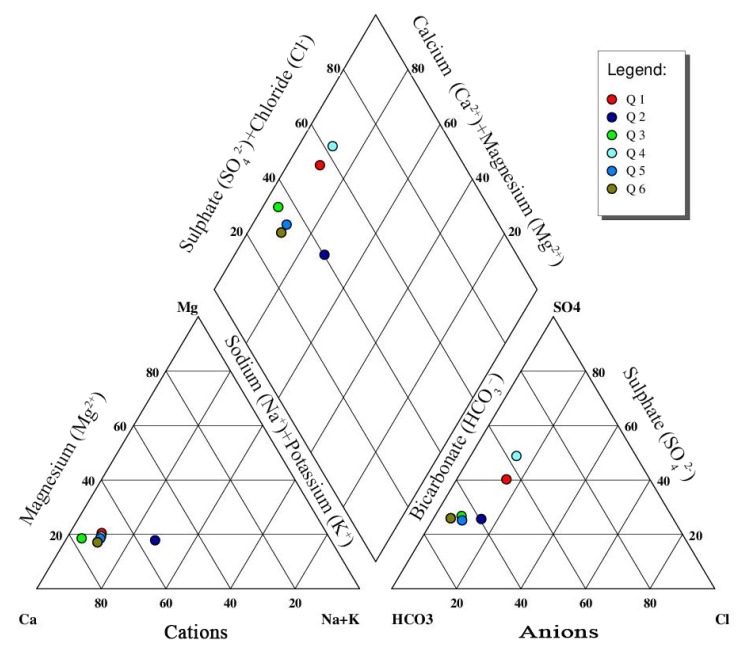
Piper trilinear diagram, describing the hydrochemical facies of the study area.

The term hydrochemical facies is a function of solution kinetics, rock-water interactions, geology, and pollution sources used to describe water quantities that differ in their chemical composition [1]. This can be clearly represented by drawing the Piper trilinear diagram [59], which is useful for geochemical evaluation among groundwater samples in more definite terms rather than with other possible plotting methods [60]. The Piper diagram consists of two triangle fields indicating the percentage distribution for the major ions to determine the hydrochemical facies of the groundwater [61]. One field for plotting cations (Ca${}^{2+}$, Mg${}^{2+}$, Na${}^{+}$, and K${}^{+}$) and the other for anions (SO${}_{4}^{2-}$, Cl${}^{-}$, and HCO${}_{3}^{-}$) [2]. The cations and anion fields are combined to show a single point in a diamond-shaped field, from which inference is drawn, based on the hydro-geochemical facies concept [10]. Aquachem 4.0 software was used to plot the Piper trilinear diagram. Figure 7 illustrates the diagram of the data obtained from the chemical analysis of the groundwater samples. The results of Piper trilinear diagram revealed the hydrochemistry types of the samples. It was found that samples Q1 and Q2 fall under the type of Ca-HCO${}_{3}$-SO${}_{4}$ and the samples Q3, Q4, Q5, and Q6 exhibited the Ca-HCO${}_{3}$ type of water. These are mainly due to the geological characteristics of the region concerned. The geological characteristics of the study area reflect the type of water from groundwater. The percentage of samples falling under the Ca-HCO${}_{3}$-SO${}_{4}$ type of water was 33.33%, while the samples falling under Ca-HCO${}_{3}$ type was 66.67%. This result indicates that the hydrochemical facies of groundwater was mostly Ca-HCO${}_{3}$ type. From the plot, it is observed that alkalis (Ca${}^{2+}$ and Mg${}^{2+}$) significantly exceeded the alkaline (Na${}^{+}$ and K${}^{+}$) and HCO${}_{3}^{-}$ exceeded other anions. The major cations in water were Ca${}^{2+}$ > Na${}^{+}$ > Mg${}^{2+}$ > K${}^{+}$, and the major anions were HCO${}_{3}^{-}$ > SO${}_{4}^{2-}$ > Cl${}^{-}$. The anions and cations in groundwater could have come basically from the dissolution of carbonate rock, and could also be associated with anthropogenic activities [62,63].

#### 3.1.3. Pearson’s Correlation Coefficient among Parameters

Correlation analysis is useful in understanding the chemical reactions occurring in the groundwater system [64]. The correlations among water quality variables can reveal several important hydrochemical relationships [65]. The Pearson’s correlation matrices were applied to identify the relationship between the variables. Correlation matrix of the 16 measured parameters was computed and presented in Table 4. Pearson’s correlation value ranges between 0 (in the case of no correlation) and 1 (when the correlation is perfect). Samples having a correlation coefficient greater than 0.7 are considered to be strongly correlated. When r takes values between 0.5 and 0.7, the samples show a moderate correlation at a significance level *p* = 0.05; while r less than 0.3 is weak [66]. From the correlation matrix, many of the physicochemical parameters showed strong correlations with each other, indicating the close association of these parameters with each other. Results of correlation analysis showed that the high positive correlations between TDS and the electrical conductivity (EC) demonstrate the increase of conductivity when the concentration of all dissolved constituents increased. It also illustrates that TDS and EC show a high positive correlation with Ca${}^{2+}$, Mg${}^{2+}$, Cl${}^{-}$, SO${}_{4}^{2-}$, NO${}_{2}^{-}$, and TH. The relationships identify clearly the main elements contributing to groundwater salinity and its tendency to follow a similar trend. High positive correlations have been reported between concentrations of Cl${}^{-}$, SO${}_{4}^{2-}$, Ca${}^{2+}$, and Mg${}^{2+}$ with correlation coefficients r taking values in the interval 0.747–0.953, thus implying the impact of the agricultural activities. Based on Table 4, correlation coefficients between Ca${}^{2+}$ and Mg${}^{2+}$ (r = 0.996 and 0.957, respectively) were statistically significant, with total hardness (TH) taking the approximate measure of Ca${}^{2+}$ and Mg${}^{2+}$. Total hardness is also correlated strongly with SO${}_{4}^{2-}$ (r = 0.918) and correlated weakly with HCO ${}_{3}^{-}$ (r = 0.439), concluding that the total hardness was essentially a permanent hardness. Concentrations of Ca${}^{2+}$ and Mg${}^{2+}$ increase when calcite and dolomite dissolve and precipitate, and HCO ${}_{3}^{-}$ decreases, leading to a higher TH and a lower TDS [67]. A strong positive correlation was observed between Ca${}^{2+}$ and Mg${}^{2+}$. It should be noted that most of the ions are involved in various physiochemical reactions [68]. The positive correlations with high values between SO${}_{4}^{2-}$ and Cl${}^{-}$ (r = 0.91), Mg${}^{2+}$ and Cl${}^{-}$ (r = 0.93), and Mg${}^{2+}$ and SO${}_{4}^{2-}$ (r = 0.95) demonstrate the impact of agricultural activity. Also, strong positive correlations between Cl${}^{-}$ and NO${}_{2}^{-}$ were significantly derived from anthropogenic-induced pollution sources, such as decaying organic matter, leakage of septic tanks, sewage wastes, and fertilizers. pH has a significant negative correlation with most of the physicochemical parameters. It should be noted that the chemical reactions inside a groundwater system incorporates various high complexities. Despite being a very effective tool, correlation analysis could only indicate the general insight into water-rock interactions [69]. If one wants to get more comprehensive and accurate results about these complex systems of water-rock interactions, there must be a complete analysis, such as aquifer mineralogy.

In line with the previous discussions, the conventional methods have provided clear information about the subsurface geologic environments in which the water is present. It also helps in determining the hydrochemical type of groundwater in relation to groundwater quality and analyzed its main pollution factors to assess the suitability of groundwater for drinking purposes. Also, with the traditional approach, the significance of the association between the different physicochemical parameters at each site of the study area is revealed. For instance, descriptive statistics, the Piper trilinear diagram, and Pearson’s correlation results are considered a necessary and indispensable tool that can be applied to distinguish the groundwater’s hydrochemical characteristics in the study area. However, there is a lack of precise information concerning the lone use of the traditional methods of water quality assessment for concise decision-making.

### 3.2. Fuzzy Logic Method for Groundwater Quality Evaluation

In this paper, fuzzy logic evaluation method was applied to assess the groundwater quality and suitability for drinking purposes in the North Chengdu Plain, according to the quality standards of (GB/T 14848-93) and the quality evaluation parameters. Based on the principles mentioned above of the evaluation technique of fuzzy logic for the groundwater quality assessment, the steps of the results of fuzzy logic can be listed in the following subsections.

#### 3.2.1. Assessment Indicators

We have assessed the quality of groundwater for six different samples (Q1, Q2, Q3, Q4, Q5, and Q6), which were obtained from the area of study. The samples mentioned above were analyzed for a number of different physicochemical water quality parameters, and nine indicators were selected from it, which include TH, TDS, NO${}_{2}^{-}$, NO${}_{3}^{-}$, NH${}_{4}^{+}$, Mn, Fe, Cl${}^{-}$, and SO${}_{4}^{2-}$. The selection considers the potential impact of these indicators on human health, as well as their water quality importance [12].

#### 3.2.2. Compute Fuzzy Matrix R

The degree of each indicator’s membership for the grade of groundwater quality was computed, according to Equation (3). Each indicator was calculated to obtain five membership levels, and the selected nine factors obtain nine numerical value sets. Hence, the matrix *R* of the fuzzy relationship is obtained from all wells selected in the area of study. Taking well Q6 as an example, the result of the fuzzy matrix *R* is as follows:

$$R=\left[\begin{array}{ccccc} 0.063 & 0.937 & 0 & 0 & 0\\ 0.565 & 0.435 & 0 & 0 & 0\\ 0.667 & 0.333 & 0 & 0 & 0\\ 0.533 & 0.467 & 0 & 0 & 0\\ 1 & 0 & 0 & 0 & 0\\ 1 & 0 & 0 & 0 & 0\\ 1 & 0 & 0 & 0 & 0\\ 1 & 0 & 0 & 0 & 0\\ 0.920 & 0.080 & 0 & 0 & 0 \end{array}\right]$$

#### 3.2.3. Weight for Each Factor

The weighting set of all wells in the study area were calculated, according to Equations (5) and (6), based on the standard (GB/T14848-93) displayed in Table 2 and data issued from Table 3 for the selected nine factors. From the example taken from well Q6, the result of weight calculation is A = (0.426, 0.185, 0.043, 0.111, 0.031, 0.008, 0.018, 0.032, 0.146).

#### 3.2.4. Fuzzy Comprehensive Evaluation

We have evaluated the fuzzy matrix *B* for the quality of groundwater evaluation, by employing the compositional process of the matrix of weight *A* and the fuzzy matrix *R*, according to Equation (7). Taking well Q6 as an instance, the result of fuzzy evaluation matrix is: B = (0.442, 0.558, 0, 0, 0). Following the maximum membership’s degree principle to obtain the level of groundwater quality, 0.558 is the maximum of the entire five values. Thus, well Q6, which fell under grade II, was categorized as good. On the other hand, the quality of the water was found to be adequate for all user purposes. Accordingly, the grades of the groundwater quality of the remaining wells in the area of study were evaluated. The corresponding results are displayed in Table 5.

According to Table 5, the fuzzy evaluation matrix B results of the remaining wells revealed that the quality of groundwater of wells Q2, Q3, and Q5, are 0.670, 0.443, and 0.429, respectively. These wells belong to class II, which is categorized as good, and their quality of water was considered suitable for various uses. The quality of groundwater corresponding to wells Q1 and Q4 were 0.568 and 0.424, respectively, and they were classified as class III, which is categorized as moderate, and their quality of water was, in general, suitable for drinking, irrigation, and suitable also in industrial production. The number of samples within the class II and III accounts for 66.67 and 33.3% of groundwater, respectively. Based on the results of the analysis of fuzzy logic method mentioned earlier, the water quality within the study area was good and suitable for drinking purposes, which indicates that fuzzy logic evaluation method can be considered as a powerful and useful tool in decision-making [28]. Fuzzy logic evaluation method considers each assessment factor’s impression on the evaluated result and determines the major pollutants based on the evaluation factors weights, and so it is more objective and scientific. In addition, using a powerful computation function in MATLAB, the fuzzy logic evaluation method is transformed into codes generating huge calculations. This assists in understanding the water quality information quickly but carefully, and providing the basis for water management. Therefore, it is a benefit for guiding the establishment of the policy of environmental protection, project development, and pollution prevention. According to the fuzzy evaluation, the indicators point to the fact that the groundwater quality of well Q1, categorized as class III, was suitable for drinking. However, it is also found that the probability of well Q1 belonging to class V was quite considerable (0.311). The observed phenomenon relating to well Q1 wes due to high concentration of the elements TH, SO${}_{4}^{2-}$, NO${}_{2}^{-}$, and NH ${}_{4}^{+}$; beyond the permissible limit. The amount of concentration in well Q1 regarding the elements can be explained by the excessive discharge of industrial and domestic sewage. By taking into account the reasons mentioned above, well Q1 was susceptible to contamination and therefore should be observed at periodic times and protected from further expected pollution. Here, it is worth mentioning that the fuzzy logic evaluation method would attain complete and precise results in evaluating groundwater quality.

The traditional methods and fuzzy logic evaluation method generated a comprehensive result for the assessment of groundwater quality in this study. The fuzzy logic evaluation method is a useful indicator of groundwater quality which overcomes the underlisted shortcomings of the traditional methods;

- Conventional methods don’t have enough flexibility when facing data set unavailability. Also, the conventional methods cannot handle the uncertainties associated with the monitoring of the quality of water.
- Traditional methods need all the suggested water quality parameters level with their guideline/standard values by considering the usage of allocated water. The main drawback of this straightforward type of assessment was its low capability of providing a holistic picture of water quality, especially for concise water quality decision-making.
- Conventional methods are based on weighted averages, but the rules of a fuzzy expert system can be built such that good quality in one parameter does not hide a lousy quality in another.

Despite the weaknesses associated with traditional methods, they are considered necessary tools to know the hydrochemical characteristics of groundwater which complements the results of more robust methods such as fuzzy logic for a comprehensive assessment of groundwater quality.

Generally, the results obtained validate the practicality and flexibility of the knowledge-based models, such as the fuzzy logic technique, for incorporating expert knowledge and modeling the current uncertainties associated with water resources and environmental perplexities. Relative to traditional methods, fuzzy logic can be used to formulate the water quality level of a region using the water quality parameters available. Given the benefits of fuzzy logic, it is suggested for complex water quality monitoring issues that are difficult to solve by conventional methods [25,38].

## 4. Conclusions

Assessment of groundwater pollution is critical for resource planning and environmental management. However, there are often no clear-cut boundaries to discriminate polluted groundwater from unpolluted groundwater. This is the situation where conventional techniques such as statistical techniques, trilinear plots, and fuzzy logic method can be used together to propose robust solutions with a high confidence level. These techniques describe the boundaries of the groundwater quality for the study samples and can be used as a broad range of environmental and natural resource management applications. In this study, these methods have been applied to provide a comprehensive assessment of the groundwater quality and its suitability for drinking purposes in the North Chengdu Plain. According to hydrochemical analysis, the groundwater in the study area was found to range between fresh and neutral in nature and was classified as very hard based on total hardness. Most of the physicochemical parameters were found within the WHO (2011) permissible limits of drinking water, except for EC, TH, TDS, Ca${}^{2+}$, and SO${}_{4}^{2-}$ in some samples. The concentrations in some samples also exceeded the (GB 5749-2006) standards. The abundance sequence of major ions is found in the order of Ca${}^{2+}$ > Na${}^{+}$ > Mg${}^{2+}$ > K${}^{+}$ and HCO${}_{3}^{-}$ > SO${}_{4}^{2-}$ > Cl${}^{-}$. A Piper diagram was used to determine hydrogeochemical types of groundwater. In general, the Piper trilinear diagram indicates that the dominant hydrochemical facies of groundwater are of Ca-HCO${}_{3}$ type, due to water–rock interaction in the study area. Statistical analysis was adopted to examine the groundwater quality variations for better understanding. A correlation analysis for major ions and pH, EC, TH, TDS, NO${}_{2}^{-}$, NO${}_{3}^{-}$, NH${}_{4}^{+}$, Mn, and Fe was used, to define the level of relationship between the hydrochemical parameters. In this study, most groups of species showed strong correlation (r > 0.7). The findings here can be associated with the concurrent increase/decrease in the cations leading to precipitation reactions and concentration effects.

A fuzzy logic evaluation method was used to determine the groundwater quality and its suitability for drinking purposes. Results achieved from this method showed that the groundwater quality of all samples were class II and III, which indicates that the quality of groundwater in all the wells was suitable for drinking. The robust nature of the analysis method in this research assures realistic evaluation of the water quality, and offers some theoretical reference to groundwater quality studies. Therefore, the method is reliable and efficient for groundwater pollution assessment, and can be used in decision-making for the optimum management of water resources, as it has globally proved to be useful in solving problems with fuzzy boundaries and controlling the effect of monitoring errors on assessment results. Hence, the proposed method is objective and practical in real applications. Considering the benefits of fuzzy logic, it is recommended to monitor complex water quality issues which are difficult to solve by traditional methods.

The integrated management of groundwater for drinking purposes is the pathway to resolving groundwater quality issues, not only in the North Chengdu Plain but also in other watersheds. Therefore, the groundwater pollution risk degree has a clear and direct connection to the discharge of water and the region’s environmental vulnerability. Moreover, in high-risk areas, wastewater treatment and management should be strengthened. The results of this study provide an essential reference frame for decision-making on improving water quality in the field of study and nearby surroundings. 

## Figures and Tables

**Table 1:** Analytical methods used in the assessment of groundwater quality.

Parameters	Analysis Methods
pH	Portable ph meter
Electrical conductivity (EC)	Portable ph meter
Total hardness (TH)	Edta complexmetry
Total dissolved solids (TDS)	Gravimetric method
Sodium (Na${}^{+}$)	Flame photometer
Potassium (K${}^{+}$)	Flame photometer
Calcium (Ca${}^{2+}$)	Volumetric methods
Magnesium (Mg${}^{2+}$)	Volumetric methods
Bicarbonates (HCO${}_{3}^{-}$)	Volumetric methods
Chlorides (Cl${}^{-}$)	Volumetric methods
Sulfates (SO${}_{4}^{2-}$)	Spectrophotometric
Nitrite (NO${}_{2}^{-}$)	Spectrophotometer
Nitrate (NO${}_{3}^{-}$)	Ionic chromatography
Ammonia (NH${}_{4}^{+}$)	Nessler is reagent spectrophotometry
Manganese (Mn)	Atomic absorption spectrophotometry
Iron (Fe)	Atomic absorption spectrophotometry

**Table 2:** Classification of groundwater quality based on the Chinese national standard (GB/T 14848-93).

Grade	Classification/Applicable Uses	Parameters								
		TH	TDS	NO_2_	NO_3_	NH_4_	Mn	Fe	Cl	SO_4_
I	Excellent suitable for drinking water	150	300	1.0	2.0	0.02	0.05	0.1	50	50
II	Good suitable for drinking water	300	500	2.0	5.0	0.02	0.05	0.2	150	150
III	Moderate suitable for drinking water	450	1000	3.0	20	0.2	3.0	0.3	250	250
IV	Poor suitable for drinking water	550	2000	10	30	0.5	10	1.5	350	350
V	Unsuitable for drinking water	>550	>2000	>10	>30	>0.5	>10	>1.5	>350	>350

**Table 3:** Descriptive statistics of groundwater parameters in comparison with WHO (2011) and Chinese national standard (GB 5749-2006).

Parameters	Units	Min	Max	Mean	Std. Deviation	WHO Guideline	National Standard
						Value (2011)	(GB 5749-2006)
pH	-	6.90	7.10	7.00	0.06	6.5–8.5	6.5–8.5
EC	μS/cm	655.93	1269.08	915.75	200.29	500	-
TH	mg/L	302.80	628.10	437.92	121.28	300	450
TDS	mg/L	387.00	824.90	566.63	146.43	500	1000
Na${}^{+}$	mg/L	10.20	58.00	25.95	17.13	200	200
K${}^{+}$	mg/L	1.80	2.50	2.25	0.25	12	-
Ca${}^{2+}$	mg/L	98.20	194.40	137.90	37.80	75	-
Mg${}^{2+}$	mg/L	13.98	34.66	22.72	6.92	50	-
HCO${}_{3}^{-}$	mg/L	146.40	411.90	307.12	95.86	500	-
Cl${}^{-}$	mg/L	12.79	78.15	36.86	22.07	250	250
SO${}_{4}^{2-}$	mg/L	85.03	276.00	140.04	71.35	250	250
NO${}_{2}^{-}$	mg/L	0.004	0.03	0.01	0.01	3	0.02
NO${}_{3}^{-}$	mg/L	0.12	3.40	1.36	1.22	50	20
NH${}_{4}^{+}$	mg/L	0.02	0.36	0.12	0.15	35	0.2
Mn	mg/L	0.003	0.19	0.05	0.07	0.1	0.05
Fe	mg/L	0.02	0.18	0.08	0.08	0.3	0.3

**Table 4:** Pearson’s correlation matrix for different groundwater quality parameters in the North Chengdu Plain.

Parameters	pH	EC	TH	TDS	Na${}^{+}$	K${}^{+}$	Ca${}^{2+}$	Mg${}^{2+}$	HCO${}_{3}^{-}$	Cl${}^{-}$	SO${}_{4}^{2-}$	NO${}_{2}^{-}$	NO${}_{3}^{-}$	NH${}_{4}^{+}$	Mn	Fe
pH	1															
EC	**−0.97**	1														
TH	**−0.85**	**0.87**	1													
TDS	**−0.95**	**0.99**	**0.91**	1												
Na${}^{+}$	**−0.29**	0.28	−0.19	0.23	1											
K${}^{+}$	−0.38	0.52	0.63	0.63	−0.01	1										
Ca${}^{2+}$	**−0.81**	**0.83**	**1.00**	**0.87**	−0.27	0.62	1									
Mg${}^{2+}$	**−0.95**	**0.97**	**0.96**	**0.99**	0.09	0.62	**0.93**	1								
HCO${}_{3}^{-}$	−0.33	0.43	0.44	0.36	−0.23	0.00	0.46	0.34	1							
Cl${}^{-}$	**−0.94**	**0.98**	**0.80**	**0.97**	0.37	0.51	**0.75**	**0.93**	0.32	1						
SO${}_{4}^{2-}$	**−0.85**	**0.90**	**0.92**	**0.93**	−0.01	0.64	**0.89**	**0.95**	0.29	**0.91**	1					
NO${}_{2}^{-}$	**−0.78**	**0.86**	**0.77**	**0.86**	0.12	0.49	**0.73**	**0.85**	0.41	**0.92**	**0.93**	1				
NO${}_{3}^{-}$	0.58	−0.44	−0.65	−0.48	0.21	−0.27	−0.66	−0.58	−0.01	−0.31	−0.40	−0.08	1			
NH${}_{4}^{+}$	−0.48	0.43	0.57	0.53	−0.05	0.60	0.55	0.60	−0.48	0.47	0.65	0.41	−0.56	1		
Mn	−0.01	0.03	0.36	0.09	−0.50	0.42	0.42	0.16	0.26	−0.16	0.01	−0.25	−0.60	0.07	1	
Fe	−0.02	−0.03	0.34	0.08	−0.47	0.46	0.38	0.17	−0.17	−0.17	0.05	−0.29	**−0.74**	0.45	**0.88**	1

Notes: **Correlation** = significant at the 0.01 level (2-tailed); Correlation = significant at the 0.05 level (2-tailed).

**Table 5:** The fuzzy evaluation of groundwater quality in the study area.

Name of Well	I	II	III	IV	V	Result Grade
Q1	0.085	0.031	**0.568**	0.005	0.311	III
Q2	0.303	**0.670**	0	0	0	II
Q3	0.064	**0.443**	0.171	0.321	0	II
Q4	0.059	0.361	**0.424**	0.156	0	III
Q5	0.225	**0.429**	0.319	0	0	II
Q6	0.442	**0.558**	0	0	0	II
